# Interpersonal stress and proinflammatory activity in emerging adults with a history of suicide risk: A pilot study

**DOI:** 10.1016/j.xjmad.2023.100016

**Published:** 2023-07-28

**Authors:** Annamarie B. Defayette, Christianne Esposito-Smythers, Ian Cero, Katherine M. Harris, Emma D. Whitmyre, Roberto López

**Affiliations:** a Department of Psychology, George Mason University, 4400 University Drive, 3F5, Fairfax, VA 22030, USA; b Department of Psychiatry, University of Rochester Medical Center, 300 Crittenden Blvd., Rochester, NY 14642, USA

**Keywords:** Interpersonal stress, Proinflammatory response, Proinflammatory cytokines, Youth, Emerging adults, Suicide risk

## Abstract

Interpersonal stress during adolescence and young adulthood can threaten healthy developmental trajectories. A “primed” proinflammatory response to acute stress may serve as an underlying process that results in negative outcomes for youth. The present pilot study examined the relation between interpersonal stress and two proinflammatory cytokines in a sample of 42 university-recruited emerging adults with recent suicidal thoughts and behaviors. Participants completed self-report measures of mood, suicidal thoughts and behaviors, recent peer-related stressors, and interpersonal sensitivity. They also participated in an acute laboratory social stress task and provided three saliva samples to measure their proinflammatory responses (IL-6 and TNF-α) to the stressor. Participants reported significant increases in sadness and exclusion, and significant decreases in inclusion, following task participation. Importantly, no participants reported an increase in or onset of suicidal thoughts. No significant associations between interpersonal stress and proinflammatory cytokines were found. Changes in affect during the task coupled with lack of increased suicidal thoughts indicate it is acceptable to use this exclusion and rejection paradigm with this population, with proper debriefing and positive mood induction procedures. Given all other nonsignificant associations, future research considerations are discussed, including impact of COVID-19 on task potency and incorporation of multiple stress response systems.

## 1 Introduction

Interpersonal stress, including loss, conflict, rejection, victimization, and maltreatment [1], can threaten healthy psychosocial developmental trajectories. Stress from peer rejection and victimization can lead to an array of negative consequences, including negative emotions, negative beliefs about the self, and mental health difficulties [2], [3]. Thus, gaining a better understanding of the processes that underlie deleterious outcomes of interpersonal stress is important. One factor directly impacted by interpersonal stress is the biological stress response. The present study examined whether peer-related interpersonal stress is associated with proinflammatory activity in a sample of university-recruited emerging adults with mental health difficulties, including suicidal thoughts and behaviors.

The biological stress response system is fundamental to reacting to an acute stressor. When a stressor is encountered, the body triggers a cascade of responses, including the “fight-or-flight” response of the autonomic nervous system (ANS) and a peripheral response which is activated by the hypothalamic-pituitary-adrenal (HPA) axis. When the HPA axis is activated, glucocorticoids are released and suppress the immune system [4]. When the HPA axis response and glucocorticoid release ends, the proinflammatory response, or the release of proinflammatory cytokines, occurs [5].

The proinflammatory response developed evolutionarily to target extracellular pathogens and initiate healing in response to physical injury [6], [7]. However, the Social Signal Transduction Theory of Depression [5] posits that a primed immunological response and subsequent release of proinflammatory cytokines can be activated in the face of perceived social threats (e.g., interpersonal stressors). This is because social threats evolutionarily had negative implications for survival (e.g., access to resources, protection). Chronic activation of the biological stress response system from interpersonal stressors can lead to glucocorticoid resistance, and in turn, upregulation of proinflammatory cytokine release. The ongoing interaction between interpersonal stress and proinflammatory activity results in sensitization to future social threats and may transfer risk for negative behavioral effects, such as depression [5].

Numerous studies offer initial support for the Social Signal Transduction Theory of Depression [5]. Studies predominantly focus on the proinflammatory cytokines interleukin-1β (IL-1β), IL-6, and tumor necrosis factor-α (TNF-α), as well as C-reactive protein (CRP; robust marker of proinflammatory activity) [5], [6], [8]. For example, a history of child maltreatment is reliably linked with elevated basal proinflammatory levels and an elevated acute proinflammatory response to psychosocial stress in clinical adult samples [9]. In studies of community-based adolescents, higher levels of stress in family, friends, and romantic relationships was associated with increased IL-6 responses to an ex vivo immune stimulation [10] and daily interpersonal stressors was positively associated with basal CRP [11].

Others have investigated this relation using a laboratory-induced performance-based social stressor, the Trier Social Stress Test (TSST) [12]. Among university-based adults, daily negative social interactions predicted elevated baseline TNF-α and elevated TNF-α and IL-6 responses to the TSST [13]. In a sample of adolescents with a history of mental health concerns, those who experienced more frequent peer victimization had elevated basal proinflammatory levels (using a latent factor comprised of IL-1β, IL-6, and TNF-α) and a heightened proinflammatory response to the TSST [8]. Cumulatively, these studies suggest that interpersonal stress, including peer-related stress, can have a prominent, lasting impact on proinflammatory activity (i.e., both basal proinflammatory levels and the proinflammatory response).

### 1.1 Current study

As is evident, interpersonal stress, including peer rejection and victimization, is associated with numerous negative consequences for adolescents and young adults. Consistent with the Social Signal Transduction Theory of Depression [5], preliminary research suggests that chronic interpersonal stress can result in an upregulated stress response. However, the extent to which these results generalize to younger samples or those with mental health difficulties, groups with heightened rates of interpersonal stress, is unclear. Additionally, while the TSST [12] is one of the most commonly used laboratory stress tasks in the current literature, it is designed and rated by youth as a *performance-based* interpersonal stressor [14]. The Yale Interpersonal Stressor (YIPS) [15], in contrast, offers an ecologically valid measure of peer exclusion and rejection, as it is designed to mimic face-to-face social exclusion and rejection. We also considered several computerized peer rejection paradigms, including Cyberball (a virtual ball tossing game with computer programmed “peers” [16], [17]) and the Chatroom Interaction Task (a virtual peer interaction paradigm where youth are led to believe that they are interacting with peers in a “chat game” [18]). Both Cyberball and the Chatroom Interaction Task have been with adolescents and adults with histories of suicidal thoughts and behaviors. Studies have primarily measured neural responses, as well as heart rate variability and serum oxytocin changes, to computerized peer rejection, and demonstrated differences in biological responses between those with versus without histories of suicidal ideation and behaviors [19], [20], [21], [22], [23]. However, no computerized peer rejection paradigm to date (i.e., at the time of study development) has been shown to elicit a salivary stress response [24]. Thus, the YIPS was the most appropriate and ecologically valid task for examining the link between peer-related stressors and proinflammatory activity in this pilot study. However, to our knowledge, the YIPS has not been used specifically with those with a history of suicide risk.

The present pilot study examined the use of the YIPS with a sample of university-recruited emerging adults with recent suicidal thoughts and behaviors. This study also examined the relation between peer-related stressors and proinflammatory activity during the YIPS. Interpersonal sensitivity, a cognitive mechanism that may underlie the association between peer victimization and negative outcomes [25], was also examined as a predictor of proinflammatory activity. We hypothesized that individuals with a greater (a) history of peer-related stressors and (b) interpersonal sensitivity would have (1) greater basal proinflammatory levels and (2) a greater proinflammatory response to the laboratory social stressor.

## 2 Methods

### 2.1 Participants

This sample included 42 emerging adults recruited from a large, diverse university in the Mid-Atlantic region of the US. Participants ranged in age from 18 to 23 years (*M* = 19.55, *SD* = 1.29). The majority of participants reported their sex assigned at birth as female (83.3 %; 16.7 % male) and identified as predominantly women (73.8 %; 16.7 % men, 9.5% nonbinary). Participants were racially diverse (45.2 % White, 16.7 % African American, 16.7 % Asian, 14.3 % multiracial, 7.1 % other) and predominantly non-Latino (88.1 %; 11.9 % Latino).

Undergraduate students enrolled in at least one class at the university were eligible to participate in the screening portion of the study. Inclusion criteria for the full study were: (1) 18–23 years of age; (2) fluent in English; and (3) recent suicidal ideation (i.e., self-reported presence of at least a wish to die within the past month at the time of eligibility screening). Exclusion criteria was a self-reported health/developmental condition that impacts immune functioning.

### 2.2 Procedures

All study procedures were approved by the university IRB prior to data collection. Data were collected between October 2020 and May 2021 and the research protocol complied with university COVID-19 health and safety requirements.

Undergraduate students were recruited from the psychology department research pool via a study ad and from the broader university community via flyers. The study was described as examining “the link between peers, stress, and mental health in young adults.” Interested students were able to self-initiate study screening using a web link via the online research pool portal or a QR code on the flyer that directed them to the screening phase of the study, completed online via Qualtrics. Students directed to the screening phase completed an electronic consent form that included a full description of the study followed by a brief eligibility screening questionnaire, which included one item that assesses for passive thoughts of death in the prior month (drawn from the Columbia Suicide Severity Rating Scale [26]; see Supplementary Material for safety protocol). Students eligible for the full study were asked to attend a laboratory visit. They completed a second consent form for the remainder of the study with a member of the research team at the start of their laboratory visit. Of note, the time between screening and the laboratory visit was delayed for a number of subjects due to scheduling limitations and COVID-19 restrictions. Half of all participants eligible at screening reported that they had not experienced thoughts of death or suicidal ideation within the past month of their laboratory visit. As this was a pilot study, these participants were retained.

The laboratory visit consisted of (1) a self-report assessment battery, (2) a laboratory social rejection task, and (3) collection of three saliva samples to measure proinflammatory cytokines. After consent was obtained, undergraduate research assistants aided participants via videoconference (i.e., Zoom) in completing an assessment battery via Qualtrics on a laboratory computer. Next, master’s level clinically trained research staff administered an interview of recent and lifetime suicidal thoughts and behaviors. Participants then completed a face-to-face peer rejection lab task and provided saliva samples, collected immediately before, immediately after, and 30 min after the task. Prior to their first sample, participants completed a 10-minute relaxation period to ensure that the measured proinflammatory response could be attributed to the task. During the relaxation period, participants listened to classical piano music and colored a mandala. After providing their first saliva sample, participants completed a pre-task questionnaire and then the lab task. Immediately after, participants completed a post-task questionnaire and provided their second saliva sample. Participants then completed a 30-minute relaxation period before providing their third saliva sample.

Once salivary sampling was completed, participants were debriefed on the true nature of the task, and then completed a positive mood induction task (to aid in the reduction of any distress induced during the laboratory visit) followed by a re-assessment of suicidal ideation (to ensure participant safety before leaving the lab). No participants reported the presence of suicidal ideation during the re-assessment. Participants were compensated with research course credits (if recruited through department research pool) or a $30 Amazon gift card.

Study visits were conducted as much as possible at a standardized time during the afternoon (3:30 PM ± 30 min) to reduce variability in salivary cytokine levels that is otherwise attributable to diurnal rhythm [27]. Most (90.48%) visits occurred during this standardized window; the rest were within 1.5 h of 3:30 PM. Participants were instructed to refrain from exercising, eating, and consuming caffeine for one hour before the laboratory visit, which may also impact salivary cytokines [13].

### 2.3 Acute laboratory stressor

The Yale Interpersonal Stressor (YIPS) [15] is a face-to-face task that mimics peer-related social exclusion and rejection to induce acute stress. Previous research has demonstrated that the YIPS induces the biological stress response in children, adolescents, and young adults [14], [15]. Participants were told they were taking part in an activity to help researchers understand “how people get to know one another” by discussing two topics (i.e., friends, weekend activities) with two “peers” for 15 min per topic. Trained undergraduate confederates served as “peers.” Past research using the YIPS has matched participants with same-sex confederates for demographic commonality [14], [15]. In the present study, participants were matched to confederates based on gender identity. Participants who identified as nonbinary were matched to confederates based on sex assigned at birth, as the research team did not have a pair of confederates who identified as nonbinary. The laboratory was arranged such that the participant and confederates were all separated by six feet (to comply with social distancing) while still facing one another, and all were required to wear face masks.

During the task, the two confederates subtly and increasingly excluded the participant through both verbal and non-verbal techniques. They made efforts to appear to bond with one another, share common interests that differed from that of the participant, and excluded the participant from conversation. Following the task, participants completed a bogus Social Perceptions Questionnaire, designed for this study to increase perceived validity of the YIPS paradigm or “story.” Items were derived from the Bogus Social Perceptions Questionnaire [18] and from an assessment of sociometric peer status for young adults [28].

### 2.4 Proinflammatory cytokines

Whole unstimulated saliva was collected via passive drool into 2 mL cryogenic vials after the 10-minute relaxation period, as well as immediately following and 30 min following completion of the YIPS. Sample collection procedures were modified due to the COVID-19 pandemic to remove in-person contact between participants and research assistants during sample collection, as well as to minimize risk of research assistant contact with saliva while still preserving the integrity of samples. A “collection kit” was placed in the participant’s data collection room prior to participant arrival. Kits contained all necessary cryovials (labeled and color-coded to correspond with each sample time point), three sets of gloves, hand sanitizer, and a small cooler that maintained a temperature of 4°C. During sample collection, research assistants instructed participants via videoconference in how to provide each sample. Each sample was placed on ice in the cooler immediately after collection and remained in the cooler for the duration of the lab visit. Immediately after the lab visit, vials were disinfected and samples were stored in a ‐20°C freezer. They were later transferred to a ‐80°C freezer for long-term storage until shipped for assay. Assays were conducted at the Institute for Interdisciplinary Salivary Bioscience Research at the University of California Irvine.

Samples were assayed in duplicate with controls for IL-6 and TNF-α (selected a priori) using proinflammatory cytokine 4-plex electrochemiluminescence immunoassays by Meso Scale Discovery (following Riis and colleagues [29]). Cytokine concentrations (pg/mL) were determined using curve fit models with MSD Discovery Workbench software. Mean lower limits of detection were 0.05 pg/mL for IL-6 and 0.04 pg/mL for TNF-α. Intra-assay coefficients of variation were 3.24 % for IL-6 % and 3.94 % for TNF-α. All IL-6 and all but one TNF-α concentrations were measurable above the lower limits of detection. The single sample with a TNF-α concentration below lower limits of detection was replaced with zero as recommended by laboratory estimates.

Descriptive statistics of raw IL-6 and TNF-α concentrations are presented in Table 1. Basal proinflammatory level corresponds to the first sample. The proinflammatory response to the YIPS was calculated using the area under the curve with respect to increase (AUCi) formula. The AUC formula is a commonly used computational approach in studies using repeated endocrinological measurements [30]. Separate AUCi values were calculated for IL-6 and TNF-α. As salivary cytokine concentration distributions are often non-normal [29], [31], [32], basal proinflammatory and AUCi concentration values were examined for normality. AUCi was calculated with raw values prior to examination of normality to preserve individual proinflammatory response patterns, as recommended by Riis and colleagues [33]. Basal IL-6 level and the IL-6 response were both skewed and leptokurtic. These variables were winsorized [33], [34] to improve normality of the distributions (see Table 2) by replacing values more extreme than ± 3 *SD* from the mean with the value equaling ± *3 SD* from the mean. This resulted in replacement of one subject’s basal IL-6 level value and one subject’s IL-6 response value. Winsorized data were used in all statistical analyses for examination of IL-6. Basal TNF-α level and the TNF-α response were normally distributed (see Table 2) and thus were not transformed.

**Table 1:** Raw proinflammatory cytokine descriptive statistics by sample time point.

Cytokine/ Time Point	*M*	*SD*	Range	Skewness (*SE*)	Kurtosis (*SE*)
IL-6					
Time 1	4.94	6.67	0.41–40.35	4.01 (0.37)	19.76 (0.72)
Time 2	4.34	4.09	0.49–20.20	1.86 (0.37)	4.65 (0.72)
Time 3	4.22	3.97	0.48–17.98	1.71 (0.37)	2.99 (0.72)
TNF-α					
Time 1	3.79	2.77	0.11–11.24	0.92 (0.37)	0.46 (0.72)
Time 2	3.18	2.58	0.00–11.11.08	1.28 (0.37)	1.70 (0.72)
Time 3	3.07	1.89	0.32–7.99	0.75 (0.37)	0.13 (0.72)

*Note.* Values are in pg/mL; Time 1 = pre-YIPS sample (basal); Time 2 = immediately post-YIPS; Time 3 = 30 min post-YIPS.

**Table 2:** Descriptive statistics of model variables.

Variable	*M*	*SD*	Range	Skewness (*SE*)	Kurtosis (*SE*)
Peer-related stressors	25.95	5.93	15–40	0.24 (0.37)	-0.14 (0.72)
Interpersonal sensitivity	97.00	14.21	66–126	-0.32 (0.37)	-0.55 (0.72)
Basal proinflammatory level					
IL-6 (winsorized)	4.58	4.85	0.41–24.95	2.50 (0.37)	7.72 (0.72)
IL-6 (raw)	4.94	6.67	0.41–40.35	4.01 (0.37)	19.76 (0.72)
TNF-α	3.79	2.77	0.11–11.24	0.92 (0.37)	0.46 (0.72)
Proinflammatory response					
IL-6 (winsorized)	-0.65	4.20	-18.16–6.98	-1.75 (0.37)	6.52 (0.72)
IL-6 (initial)^a^	-0.96	5.74	-31.33–6.98	-3.69 (0.37)	19.19 (0.72)
TNF-α	-0.97	2.59	-7.49–4.60	-0.37 (0.37)	0.75 (0.72)

*Note.* Proinflammatory values are in pg/mL.

a IL-6 AUCi value prior to being winsorized.

### 2.5 Measures

#### 2.5.1 Columbia suicide severity rating scale (C-SSRS)

The C-SSRS [26] is a self-report measure that assesses a broad spectrum of suicidal thoughts and behaviors, as well as non-suicidal self-injury. The C-SSRS measures four constructs: intensity of suicidal ideation; severity of suicidal ideation; suicide attempt (aborted, interrupted, actual); and lethality of any attempts [35]. The C-SSRS Baseline/Screening Version, which accounts for both lifetime suicidal thoughts and behaviors as well as those that have occurred within a given “current” time frame (“past month” in the present study). The intensity of ideation construct was re-administered at the end of the visit to ensure no significant increase in suicidal ideation occurred during the study visit.

#### 2.5.2 Depression anxiety stress scales (DASS-21)

The DASS-21 [36], [37] is a self-report measure that assesses the negative emotion states of depression, anxiety, and stress (e.g., “I felt that I had nothing to look forward to”, “I found myself getting upset rather easily”). The DASS-21 consists of 21 items rated on a 4-point Likert type scale (0 = “did not apply to me at all,” 3 = “applied to me very much, or most of the time”). There are three scales that correspond to the three negative emotion states. A total score can also be calculated. The DASS-21 has demonstrated satisfactory to good reliability and adequate construct validity [36].

#### 2.5.3 Affect ratings

Participants provided affect ratings immediately before and immediately after the YIPS to assess whether the task evoked perceived changes in affect and feelings of inclusion and exclusion. Participants reported how strongly they felt a series of emotions at that moment (happy, sad, angry, nervous, included, excluded, lonely, and hopeless), on a 0 (“not at all”) to 10 (“very strongly”) Likert-type scale. Items of “lonely” and “hopeless” were selected based on their strong association with suicidal thoughts and behaviors [38], [39]; the remaining items were selected from affect ratings used in other lab stressor tasks [18].

#### 2.5.4 Responses to stress questionnaire – Peer social stress version (RSQ)

The RSQ Peer Stress (College Student) version [40] is a self-report measure comprised of a checklist of recent peer stress events, three coping strategy scales, and two involuntary stress response scales. In the present study, only the total peer-related stressors score from the 15-item checklist of events was used. Participants rated the degree to which each event has been stressful for them over the past six months (e.g., “someone spreading rumors about you”, “not being invited to do things with others”) using a 4-point Likert-type scale (1 = “not at all,” 4 = “very”).

#### 2.5.5 Interpersonal sensitivity measure (IPSM)

The IPSM [41] is a self-report measure that assesses heightened sensitivity to interpersonal interactions and negative evaluations by others (e.g., “I avoid saying what I think for fear of being rejected”). The IPSM consists of 36 items rated on a 4-point Likert-type scale (1 = “not at all like me,” 4 = “very like me”) and includes five subscales and a total score. The IPSM has demonstrated good internal consistency in clinical and non-clinical samples [41], [42].

#### 2.5.6 Health factors survey

Consistent with past studies [29], [31], [32], participants reported on several health factors that may influence proinflammatory cytokines. Oral health questions included smoking status, frequency of brushing teeth, and any current oral infections. Systemic health questions included current medication use, past 48-hour alcohol/substance use, and presence/absence of a current cold or illness (e.g., cough, sinus infection, fever). Height and weight were measured in the lab to calculate BMI.

### 2.6 Data analysis plan

Power estimates were conducted in R [43] with the RStudio development environment [44]. *A priori* analysis suggested a sample size of *N* = 50 would be sufficient to detect an effect of *r* = 0.40 at power (1-β) = 0.81. Given time and resource constraints, a final sample size of *N* = 42 was recruited. To evaluate how well this sample addresses the research question above, we simulated 100 multivariate normal samples with *k* = 8 variables each (4 outcomes and 2 predictors per sample, all equally correlated with one another; see Supplementary material for full R code). We varied the strength of the correlation between variables in the simulated data, then analyzed them with the regression model described in the methods, keeping track of the results for each simulated sample to estimate overall power. Results of this analysis showed that the current sample is capable of detecting a single small effect (*r* = 0.25) with 1-β = 0.35. Although this power is low, we conducted several pairs of independent tests on the same question That is, for each of the 4 outcome variables, there are 2 predictors capable of manifesting a significant effect, if there a true effect of *r* = 0.25 is to be found. Simulations showed the power to detect at least one significant effect per outcome was much higher (1-β = 0.56). Finally, assuming a true effect of *r* = 0.25, the power to detect at least one significant effect in this study was 1-β = 0.96.

All analyses were conducted using SPSS Version 28 [45]. Descriptive statistics and distributional properties of all study variables, as well as correlations between variables of interest and possible covariates, were examined. One participant was missing all pre-task affect rating data due to administration error and thus was dropped from analyses of affect rating changes. Three additional participants were missing pre- and post-task affect rating data specifically for ratings of “lonely” and “hopeless” and were therefore excluded specifically from analyses of changes in loneliness and hopelessness. No other data were missing across any variables of interest. Thus, the complete sample was included in all preliminary and primary analyses.

Changes in self-reported affect ratings from before and after the YIPS were examined using paired samples t-tests. To examine study hypotheses, a series of hierarchical multiple regression analyses were conducted. For each model, relevant covariates were entered into step 1 and the predictor was entered into step 2. Predictor variables were the RSQ total peer stress score (peer-related stressors) and IPSM total score (interpersonal sensitivity). IL-6 and TNF-α were examined separately. To examine Hypothesis 1, basal proinflammatory level was regressed onto one of the two predictor variables. To examine Hypothesis 2, proinflammatory response was regressed onto one of the two predictor variables. All predictors were mean centered.

## 3 Results

### 3.1 Descriptive statistics

Descriptive statistics and bivariate correlations among all model variables are presented in Table 2, Table 4, respectively. Clinical characteristics of the sample, drawn from the C-SSRS and DASS-21, are presented in Table 3. The sample had high rates of internalizing symptoms, with 57.14% and 64.29% of the sample falling in the moderate-extremely severe ranges for symptoms of depression (score of 14 or greater) and anxiety (score of 10 or greater) [37], respectively. Additionally, 26.2% of participants reported current psychiatric medication use.

**Table 3:** Clinical characteristics of the study sample.

			*n* (%)	Range	*M*	*SD*
**Symptoms of Depression/Anxiety**						
Depression			--	0–38	16.57	9.76
Anxiety			--	0–28	11.76	6.85
**Most Severe Suicidal Ideation**						
Lifetime			--	1–5	3.14	1.51
Past Month			--	0–3	0.76	0.88
**Lifetime Suicidal Behavior**						
Actual suicide attempt						
	No		33 (78.6)	--	--	--
	Yes		9 (21.4)	--	--	--
		Number of actual attempts*	--	1–6	2.33	1.73
Interrupted suicide attempt						
	No		37 (88.1)	--	--	--
	Yes		5 (11.9)	--	--	--
		Number of interrupted attempts*	--	1–11	3.8	4.21
Aborted suicide attempt						
	No		33 (78.6)	--	--	--
	Yes		9 (21.4)	--	--	--
		Number of aborted attempts^* †^	--	1–112	13.78	36.84
Preparatory acts or behavior						
	No		29 (69.0)	--	--	--
	Yes		13 (31.0)	--	--	--
**Non-Suicidal Self-Injury**						
Lifetime						
	No		17 (40.5)	--	--	--
	Yes		25 (59.5)	--	--	--
Past 3 months						
	No		37 (88.1)	--	--	--
	Yes		5 (11.9)	--	--	--

*Note.* Clinical characteristics presented are based on responses to the DASS-21 depression and anxiety scales and the C-SSRS.

*Descriptive statistics associated with number of attempts are based on participants who responded “yes” to a lifetime attempt.

^†^Median number of aborted attempts = 9.

**Table 4:** Correlations among study variables and examined covariates.

	1.	2.	3.	4.	5.	6.	7.	8.	9.	10.	11.	12.	13.	14.
1. Age	---													
2. Sex (at birth)^a^	-.16	---												
3. Depressive symptoms	-0.06	0.11	---											
4. Anxiety symptoms	0.00	0.14	0.61**	---										
5. BMI	-0.04	0.24	-0.15	0.06	---									
6. Cigarette use^b^	.02	0.16	-0.05	0.27	0.14	---								
7. Alcohol/substance use^c^	.23	-0.08	-0.21	-0.21	-0.08	-0.01	---							
8. Average teeth brushing/day	-0.35*	.13	-0.07	-0.35*	-.18	-0.09	-0.11	---						
9. Use of psychiatric medication^d^	.00	0.12	0.29	0.20	0.07	-0.05	-0.05	0.16	---					
10. Basal IL-6 level^e^	.12	0.11	0.07	0.06	0.32_*_	.04	-0.14	-0.05	0.10	---				
11. Basal TNF-α level	0.01	-0.05	0.04	0.00	0.05	-0.15	-0.28	-0.07	-0.17	0.52**	---			
12. IL-6 response^e^	.20	-0.19	-0.14	-0.23	-0.20	-0.12	0.27	0.15	-0.16	-0.73**	-.40**	---		
13. TNF-α response	0.16	-0.09	-0.13	-0.08	-0.17	-0.04	0.31*	.09	0.07	-0.21	-0.63**	.44**	---	
14. Peer-related stressors	-0.10	0.12	0.27	0.39*	.05	0.29	-0.15	-0.20	-0.13	0.06	0.09	-0.15	-0.22	---
15. Interpersonal sensitivity	-0.26	0.01	0.45**	.46**	-.11	-0.13	-0.14	-0.04	0.19	-0.14	0.15	-0.18	-0.26	0.41**

a 0 = male and 1 = female;

b 0 = no and 1 = yes (less than once/month);

c 0 = no and 1 = yes (in the past 48 h);

d 0 = no and 1 = yes;

e IL-6 values are winsorized.

* *p* < .05;

** *p* < .01.

### 3.2 Affective responses

Results of all paired samples t-tests examining pre- to post-task changes in affect ratings are presented in Table 5. Results suggested that the YIPS evoked significant increases in feelings of sadness, *t*(40) = 2.17, *p* = .04 (pre-task *M* = 2.10, *SD* = 2.26; post-task *M* = 2.41, *SD* = 2.42) and exclusion, *t*(40) = 7.90, *p* < .001 (pre-task *M* = 1.00, *SD* = 1.83; post-task *M* = 4.73, *SD* =;3.16), and a significant decrease in feelings of inclusion, *t*(40) = −4.43, *p* < .001 (pre-task *M* = 4.49, *SD* = 2.94; post-task *M* = 2.54, *SD* = 2.23).

**Table 5:** Pre- to Post-YIPS changes in affective responses.

Affect Item	Pre-YIPS Rating		Post-YIPS Rating		N	*t*	df	*p*	*d*
	*M*	*SD*	*M*	*SD*					
happy	4.56	2.32	4.02	2.23	41	-1.73	40	0.09	-0.27
sad	2.10	2.26	2.41	2.42	41	2.17*	40	0.04	0.34
angry	0.51	1.31	0.61	1.24	41	0.49	40	0.63	0.08
nervous	3.15	3.02	3.24	2.76	41	0.24	40	0.81	0.04
included	4.49	2.94	2.54	2.23	41	-4.43**	40	< 0.001	-0.69
excluded	1.00	1.83	4.73	3.16	41	7.90**	40	< 0.001	1.23
distressed	2.34	2.69	2.29	2.39	41	-0.11	40	0.92	-0.02
lonely	2.66	2.61	3.00	2.55	38	0.92	37	0.36	0.15
hopeless	1.47	2.28	1.34	2.33	38	-0.64	37	0.53	-0.10

* *p* < .05.

** *p* < .001.

### 3.3 Covariates

Possible covariates examined were age, sex at birth, depressive symptoms, anxiety symptoms, and health factors. Health factors included BMI, average frequency of brushing teeth per day, smoking status, presence of an oral infection, presence of a cold/illness, past 48-hour alcohol/substance use, and current psychiatric medication use (all “yes” or “no”). No participants reported presence of an oral infection or of a cold/illness, so these variables were not considered further. At the bivariate level, significant associations were found between: BMI and basal IL-6 level; and alcohol/substance use and TNF-α response (see Table 4). No other potential covariates were associated with any of the proinflammatory activity outcome variables (*p*s > 0.05). For consistency, BMI was included as a covariate in all models of IL-6 activity and alcohol/substance use was included as a covariate in all models of TNF-α activity.

### 3.4 Main hypotheses

Results of all hierarchical multiple regression models are presented in Table 6. Peer-related stressors was not associated with basal IL-6 level (β = 0.04, *p* = .78) or basal TNF-α level (β = 0.04, *p* = .78). Interpersonal sensitivity was also not associated with basal IL-6 level (β = −0.11, *p* = .48) or basal TNF-α level (β = 0.11, *p* = .48).

**Table 6:** Hierarchical multiple regression models predicting proinflammatory activity.

Model	Basal proinflammatory activity					Proinflammatory response				
	*B*	*SE B*	β	*p*	R^2^/ΔR^2^^d^	*B*	*SE B*	β	*p*	R^2^/ΔR^2^^d^
**Models Predicting IL-6**										
*Covariate*^a^										
BMI	0.25	0.12	0.32	0.04*	.10	-0.13	0.11	-0.20	0.21	0.04
*Predictor*^b^										
Peer-related stressors	0.04	0.12	0.04	0.78	0.002	-0.10	0.11	-0.14	0.37	0.02
Interpersonal sensitivity	-0.04	0.05	-0.11	0.48	0.01	-0.06	0.05	-0.21	0.19	0.04
**Models Predicting TNF-α**										
*Covariate*^a^										
Alcohol/substance use^c^	-1.86	1.01	-0.28	0.07	0.08	1.94	0.94	0.31	0.045*	.10
*Predictor*^b^										
Peer-related stressors	0.02	0.07	0.04	0.78	0.002	-0.08	0.07	-0.17	0.26	0.03
Interpersonal sensitivity	0.02	0.03	0.11	0.48	0.01	-0.04	0.03	-0.22	0.15	0.05

a Covariate entered into step 1 of each model;

b predictors were examined in separate models;

c 0 = no and 1 = yes (in the past 48 h).

d Because each model included only one predictor at each step, R^2^ (step 1) and ΔR^2^ (step 2) also represent the individual effect size of the corresponding predictor.

* *p* < .05.

Similarly, peer-related stressors was not associated with the IL-6 response (β = −0.14, *p* = .37) or the TNF-α response (β = −0.17, *p* = .26). Interpersonal sensitivity was also not associated with the IL-6 response (β = −0.21, *p* = .19) or the TNF-α response (β = −0.22, *p* = .15). In sum, neither recent history of peer-related stressors nor interpersonal sensitivity predicted either basal proinflammatory levels or the proinflammatory response to the interpersonal stressor.

## 4 Discussion

In this pilot study, we sought to extend (1) the use of the Yale Interpersonal Stressor [15] and (2) the Social Signal Transduction Theory of Depression [5] in an examination of the relation between peer-related stressors, as well as interpersonal sensitivity, and proinflammatory activity (IL-6 and TNF-α cytokines). To our knowledge, this is the first study to use the YIPS as an acute laboratory stressor in a sample of young adults with a history of suicidal thoughts and behaviors. Our findings suggested that participants felt significant increases in sadness and exclusion, and a significant decrease in inclusion, following the YIPS. This is consistent with the goal of the YIPS, which is to mimic face-to-face social exclusion and rejection [15]. Given the nature of the present sample, at the end of the research visit all participants were debriefed on the true purpose of the task and completed a positive mood induction followed by a re-assessment for any current suicidal ideation. Importantly, no participants reported an increase in or onset of suicidal thoughts during the re-assessment. We cannot rule out the possibility that fleeting suicidal thoughts arose briefly between task completion and the end of the positive mood induction. However, if this were the case for any participants, the thoughts either did not last beyond debriefing and the positive mood induction, and/or the experience was not reported during re-assessment. This pilot study therefore provides support for the use of the YIPS, along with proper debriefing and positive mood induction procedures, in this population of emerging adults with a history of suicidal thoughts and behaviors. Notably, the present findings should *not* be generalized to samples of emerging adults with a higher level of current suicidal ideation severity or clinical acuity.

Contrary to study hypotheses, no statistically significant association was found between peer-related stressors or interpersonal sensitivity and either basal proinflammatory levels or the proinflammatory response. Findings related to recent history of peer-related stressors are contrary to prior research demonstrating positive associations between peer stress and both IL-6 and TNF-α [8], [10], [13], but are consistent with several studies finding mixed support for this hypothesized association, highlighting the degree of variability in the current literature on the proinflammatory response.

### 4.1 Interpretive considerations

Several interpretive caveats should be considered in this pilot study. First, the present study may not have captured the full stress response and recovery window. Salivary sampling was limited to three time points and captured 30 min post-stressor. As this was a pilot study, this decision was partially driven by budgetary constraints. Optimal sampling timing for examining the proinflammatory response to stress is, as of yet, not fully determined, and studies range from only two sampling points to multiple points collected across two hours post-stressor [46].

Second, laboratory visits were conducted during the COVID-19 pandemic, which may have impacted the lab task and associated findings. To maximize safety of participants and research staff alike, the lab task was modified to include mask wearing and social distancing. Masks limit facial feedback that can be important for interpreting nonverbal cues during social interactions, and may impact a person’s ability to properly hear what is said by others. Additionally, social distancing may have decreased the intimacy and impact of the social interaction. While affective changes (sadness, exclusion, and inclusion) were reported pre-to-post task, it is possible that these COVID-19 related changes reduced the potency of the YIPS and thus the likelihood of a biological response. Nonetheless, use of a modified in-person stressor task, rather than use of a virtual alternative was deemed preferable because of the body of evidence demonstrating a lack of neuroendocrine response to virtual stress tasks [47], [48]. It is also highly likely that participants experienced social isolation for many months prior to their participation in this study, impacting reports of both recent histories of peer-related stressors and sensitivity to interpersonal interactions. In sum, the full extent of the impact of COVID-19 on the present study cannot be determined.

Several additional limitations exist, including sample size and composition, as well as medication use. Specifically, first, as this was a pilot study, the sample was limited in size. Although power to detect a single small effect in the present study was lower, power simulations suggested that the present study was adequately powered to detect at least one significant effect across analyses (see Supplementary Material). Notably, estimation of power analysis parameters are particularly difficult to determine for proinflammatory cytokines because population-level cytokine concentration norms and ranges are not established [33]. Second, this study did not include a control group of participants with no lifetime history of suicidal thoughts and behaviors, which limits the extent to which conclusions can be drawn. Third, the sample was disproportionately comprised of cisgender females and we were unable to examine group differences by gender or sexual identity due to very small cell sizes. Additionally, the sample only included emerging adults who were actively enrolled in at least one university course, which limits generalizability. Emerging adults with similar demographic characteristics who do not attend university may differ from their university-enrolled counterparts in ways relevant to the theory underlying this study. For example, youth who have early adverse experiences are less likely to attend and complete college [49], [50], [51]. As early adverse experiences often include experiences of interpersonal stress or result in interpersonal stress, the present sample does not capture a subset of emerging adults whose experiences are relevant for understanding the link between interpersonal stress and proinflammatory activity. Last, approximately one quarter of participants also reported psychiatric medication use, which has been shown to impact proinflammatory cytokines [52]. However, medication use was not associated with any proinflammatory cytokine variables at the bivariate level in this study.

### 4.2 Future directions

Future work should consider the use of more complex biopsychosocial models of stress responsivity that incorporate developmental factors and multiple stress response systems, as is common in the area of developmental psychopathology [53]. The Social Signal Transduction Theory of Depression is predicated on the assumption that a transaction between interpersonal stress and activation of proinflammatory cytokines occurs across an extended period of time [8]. Thus, important developmental factors for future work include presence and chronicity of experiences of childhood maltreatment, peer victimization, and interpersonal trauma, which are also known risk factors for suicidal thoughts and behaviors [54].

Additionally, an emerging body of literature is examining the importance of coordination of the HPA axis, ANS, and inflammatory systems, as well as the relative impact of asymmetrical stress response coordination on mental health outcomes [52], [55], [56]. For instance, glucocorticoid resistance and subsequent upregulation of proinflammatory activity is an example of when the HPA axis and inflammatory system are no longer properly coordinated. Limiting examination to a single aspect of the stress response, such as in the present study, may mask the true nature of effects.

### 4.3 Conclusion

This study sought to examine the relation between peer-related stressors, as well as interpersonal sensitivity, and proinflammatory activity among university-recruited emerging adults with a recent history of suicidal thoughts and behaviors. While no associations between interpersonal stress and proinflammatory cytokines were found, subjective reports of changes in affect coupled with lack of increased suicidal thoughts during the task suggest that, with proper debriefing and positive mood induction procedures, the YIPS is acceptable in this population of emerging adults with a recent history of suicidal thoughts and behaviors. However, this should not be generalized to emerging adults at higher clinical acuity from the present findings alone. Future research should consider incorporating developmental factors and coordination of multiple stress response systems into more complex models of responsivity to interpersonal stress.

## Ethics Approval

This study was approved by the university IRB prior to data collection.

## Patient Consent Statement

All subjects provided informed consent prior to study participation.

## Declaration of Competing Interest

The authors declare the following financial interests/personal relationships which may be considered as potential competing interests: Annamarie Bailey Defayette reports financial support was provided by National Institute of Mental Health. Annamarie Bailey Defayette reports financial support was provided by American Psychological Foundation. Annamarie Bailey Defayette reports financial support was provided by Psi Chi International Honor Society in Psychology. Annamarie Bailey Defayette reports financial support was provided by American Psychological Association.

## Data Availability

The data that support the findings of this study are available from the corresponding author upon reasonable request
