# Supplementary material for: Interpersonal stress and proinflammatory activity in emerging adults with a history of suicide risk: A pilot study
Source: J Mood Anxiety Disord. 2023 Jul 28;2:100016. doi: 10.1016/j.xjmad.2023.100016 (PMC10486198; doi:10.1016/j.xjmad.2023.100016)
Supplement: Supplementary file 1 — Supplementary material [file mmc1.docx]

**Supplementary Material**

**Safety Protocol**

The safety protocol for the present study differed by study phase. This protocol was developed in partnership with the Student Support and Advocacy Center (SSAC) at the university where data collection occurred and approved by the university IRB.

*Eligibility Screening*

Responses to the eligibility screening were reviewed every 24 hours, paying particular attention to the suicidal ideation item (“In the past month, I wished I was dead or wished I could go to sleep and not wake up”). Participants who were eligible for the full study were those who responded positively to this statement (i.e., *rarely*, *sometimes*, *often*, or *always*). These participants were contacted immediately to schedule their laboratory visit, where more thorough assessment for suicidality occurred. Eligible screening participants who responded *often* or *always* to the suicidal ideation item were referred to SSAC for assistance with connecting to support services immediately after their screening survey was received; those who responded *rarely* or *sometimes* were referred to SSAC if they did not respond to lab visit scheduling emails within two weeks of their screening survey completion. Screening participants who either were eligible but chose to not continue with the full study or who met other exclusion criteria (but responded positively to the suicidal ideation item) were also referred to SSAC. All screening participants were made aware that they could be referred to SSAC at the time of consent. All screening participants referred to SSAC were also provided with a list of therapeutic support services and crisis resources via email.

*Laboratory Visit*

The laboratory visit safety protocol was designed in consultation with the Columbia Protocol triage and risk identification guidelines for emergency departments [1]. This protocol and procedure were designed by suicide experts at Columbia University and is considered the gold standard in suicide risk assessment, used widely in research and clinical practice across the United States. The Columbia Suicide Risk Severity Scale (C-SSRS) [2] was used to assess suicidal ideation over the past month (as well as lifetime) and suicidal behaviors over the past three months (as well as lifetime) during the laboratory visit. A *severity of ideation* score is derived based on five items corresponding to thoughts about death (score = 1), suicidal ideation (score = 2), thoughts about a suicide plan (score = 3), suicidal intent (to follow through on suicidal thoughts; score = 4), and suicide plan (score = 5). Suicidal behavior corresponds to a report of preparatory acts toward a plan or a suicide attempt.

Participants who endorse any *severity of ideation* item on the C-SSRS (score = 1-5) or any suicidal behavior on the C-SSRS were referred to SSAC, as well as provided with resources for support services. Of note, any participants who were referred to SSAC during the screening phase (prior to attending their lab visit) were not referred a second time; however, for these participants, SSAC was provided with confirmation of their lab visit attendance and any notable additional information from the C-SSRS (e.g., history of suicidal behavior). Further, consistent with the C-SSRS triage guidelines, participants whose highest *severity of ideation* score was a 3, 4, or 5 on the C-SSRS (corresponding to suicidal ideation with some thoughts about a suicide plan and/or intent) and/or (as outlined prior to data collection) who reported suicidal behavior (corresponding to an actual, interrupted, or aborted attempt, or preparatory acts or behavior) in the past three months received a “warm handoff call” to the university Counseling and Psychological Services (CAPS; i.e., study staff called CAPS with a referral for the participant) with the participant present. During this call, the CAPS clinician conducted a brief assessment to determine if more immediate assessment is needed (e.g., by Mobile Crisis). The protocol as outlined prior to data collection was also such that in the event of suicidal behavior in the past 24 hours, regardless of what was endorsed on the C-SSRS, a call to emergency services (Mobile Crisis, 911) would be made; however, no participants endorsed past 24-hour suicidal behavior. All laboratory visits were scheduled while CAPS was open.

**Power Estimates**

*Simulated Power Estimates in R*

Power estimates are produced via simulation of multivariate normal datasets.

We begin by setting the seed for reproducible results.

library(tidyverse)

## ── Attaching packages ─────────────────────────────────────── tidyverse 1.3.2 ──
## ✔ ggplot2 3.3.6 ✔ purrr 0.3.4
## ✔ tibble 3.1.8 ✔ dplyr 1.0.9
## ✔ tidyr 1.2.0 ✔ stringr 1.4.1
## ✔ readr 2.1.2 ✔ forcats 0.5.2
## ── Conflicts ────────────────────────────────────────── tidyverse_conflicts() ──
## ✖ dplyr::filter() masks stats::filter()
## ✖ dplyr::lag() masks stats::lag()

set.seed(107260)

Create a function for generating multivariate data with a particular number of predictors, outcomes, and correlation between them (r). All variable correlations are the same.

generate_sample <- function(n, r, outs, preds) {
 sigma <- diag(nrow = outs + preds)
 sigma[sigma != 1] <- r

 var_names <- c(
 paste0('y', 1:outs),
 paste0('x', 1:preds))

 mvtnorm::rmvnorm(
 n = n,
 sigma = sigma) %>%
 as.data.frame() %>%
 set_names(var_names) %>%
 mutate(subject_id = row_number()) %>%
 select(subject_id, everything())
}

We then use the sample generating function to simulate data for a range of correlations (i.e., r = .10 to .50), with 100 generated samples per population correlation.

sim_data <- expand_grid(
 samp = 1:100,
 r = seq(.10, .50, by = .05)) %>%
 group_by(samp, r) %>%
 mutate(
 data = map(r, ~ generate_sample(n = 42, r = .x, outs = 4, preds = 2))) %>%
 unnest(data)

With a small amount of data wrangling, we can then create a dataset representing a simulated collection of samples. We pivot the dataset in such a way that we can quickly make the sample easily analyzable.

pivoted_sim_data <- sim_data %>%
 group_by(samp, r, subject_id) %>%
 pivot_longer(
 cols = starts_with('x'),
 names_to = 'pred_var',
 values_to = 'pred_val') %>%
 pivot_longer(
 cols = starts_with('y'),
 names_to = 'out_var',
 values_to = 'out_val') %>%
 group_by(samp, r, pred_var, out_var) %>%
 nest()

We then fit the study’s analysis model to each simulated sample, keeping track of the results in each one.

pivoted_sim_results <- pivoted_sim_data %>%
 ungroup() %>%
 mutate(
 fit = map(.x = data, .f = ~ lm(out_val ~ pred_val, data = .x)),
 model_stats = map(.x = fit, .f = ~ broom::glance(.x))) %>%
 unnest(model_stats) %>%
 select(samp, r, out_var, pred_var, r.squared, p.value)

Power estimates for one single effect

pivoted_sim_results %>%
 group_by(r) %>%
 filter(out_var == 'y1' & pred_var == 'x1') %>%
 summarise(one_test_power = mean(p.value < .05))

## # A tibble: 9 × 2
## r one_test_power
## <dbl> <dbl>
## 1 0.1 0.13
## 2 0.15 0.19
## 3 0.2 0.22
## 4 0.25 0.35
## 5 0.3 0.61
## 6 0.35 0.67
## 7 0.4 0.72
## 8 0.45 0.79
## 9 0.5 0.95

Power estimates for at least one significant effect per outcome

pivoted_sim_results %>%
 group_by(samp, r, out_var) %>%
 summarise(any_preds_sig = any(p.value < .05), n = n()) %>%
 filter(out_var == 'y1') %>%
 group_by(r) %>%
 summarise(two_pred_power = mean(any_preds_sig))

## `summarise()` has grouped output by 'samp', 'r'. You can override using the
## `.groups` argument.

## # A tibble: 9 × 2
## r two_pred_power
## <dbl> <dbl>
## 1 0.1 0.18
## 2 0.15 0.32
## 3 0.2 0.45
## 4 0.25 0.56
## 5 0.3 0.82
## 6 0.35 0.82
## 7 0.4 0.87
## 8 0.45 0.98
## 9 0.5 1

Power estimates for at least one significant effect across entire study

pivoted_sim_results %>%
 group_by(samp, r) %>%
 summarise(any_preds_sig = any(p.value < .05), n = n()) %>%
 group_by(r) %>%
 summarise(eight_pred_power = mean(any_preds_sig))

## `summarise()` has grouped output by 'samp'. You can override using the
## `.groups` argument.

## # A tibble: 9 × 2
## r eight_pred_power
## <dbl> <dbl>
## 1 0.1 0.52
## 2 0.15 0.77
## 3 0.2 0.86
## 4 0.25 0.96
## 5 0.3 0.98
## 6 0.35 0.98
## 7 0.4 1
## 8 0.45 1
## 9 0.5 1

**References**

[1] The Columbia Lighthouse Project, Columbia Lighthouse Proj. (n.d.). https://cssrs.columbia.edu/ (accessed May 9, 2023).

[2] K. Posner, D. Brent, C. Lucas, M. Gould, B. Stanley, G. Brown, J. Zelazny, P. Fisher, A. Burke, M. Oquendo, J. Mann, Columbia-Suicide Severity Rating Scale (C-SSRS), (2009). https://cssrs.columbia.edu/wp-content/uploads/C-SSRS1-14-09-Baseline.pdf.
